# Supplementary material for: Identification and expression pattern of chemosensory genes in the transcriptome of Propsilocerus akamusi
Source: PeerJ. 2020 Jul 21;8:e9584. doi: 10.7717/peerj.9584 (PMC7380273; doi:10.7717/peerj.9584)
Supplement: Supplemental Information 3 [file peerj-08-9584-s003.docx]

Table S1. Primers used for RT-qPCR analysis of olfactory genes of the *P. akamusi*

| Gene | Sequence | Length(bp) |
| --- | --- | --- |
| Odorant binding proteins | |  |
| OBP1 | Sense: AGACGGGAATTAGCAGA | 165 |
|  | Antisense: CAGCAACTAGACGAGCAA |  |
| OBP2 | Sense: CTGACGTGGACAGAATAG | 138 |
|  | Antisense: TTGCCAATGACATGAAAC |  |
| OBP3 | Sense: GCTACCGTTGATGCTTG | 133 |
|  | Antisense: CGGCTGTTGGTTTCG |  |
| OBP4 | Sense: AAGCTGGATTTATGGATG | 130 |
|  | Antisense: GGTGTTGCTCCCTTTT |  |
| OBP5 | Sense: GCTGCATGGGCTCCGATAA | 131 |
|  | Antisense: TTGCCAATGAGACCAACTTCC |  |
| OBP6 | Sense: AGAGGGAAAGTGCTTATT | 104 |
|  | Antisense: CAGACATTGTCGAGGC |  |
| OBP7 | Sense: ATGCAAGTGCCGAGAA | 128 |
|  | Antisense: CACCTCCATCTGTGGTTC |  |
| OBP8 | Sense: GGCGGTTTGGTTGAT | 147 |
|  | Antisense: TTTGCCGATTTGTGC |  |
| OBP9 | Sense: TTCGGAGTAACCAAAGA | 107 |
|  | Antisense: GCAATATCATCAGAGGC |  |
| OBP10 | Sense: TGCTTCAACCACCAG | 195 |
|  | Antisense: CAGGGTCCTCAACATC |  |
| OBP11 | Sense: CTAAAACAGGAATCGA | 170 |
|  | Antisense: TTTAAGCCTTTCACCT |  |
| OBP12 | Sense: GGCAATCGCAAAATA | 164 |
|  | Antisense: ATCACCCCAAATCCT |  |
| OBP13 | Sense: ATGGATGAGGAAGGC | 140 |
|  | Antisense: GATTTATTGCAGATTGT |  |
| Odorant receptors | |  |
| OR1 | Sense: ATCAAGCGTATGAAGGA | 170 |
|  | Antisense: AATGGCGTTGGAGTG |  |
| OR2 | Sense: TGCCGTCATCTCAATC | 103 |
|  | Antisense: TAAGTAAGCACCCGTTT |  |
| OR3 | Sense: TTTCGGAGCATTTCAG | 175 |
|  | Antisense: CATTCGGATTGCTGAT |  |
| OR4 | Sense: ATCCGAAAATAAAACG | 144 |
|  | Antisense: GTAAAACTGTGCAGTAAGA |  |
| OR5 | Sense: AATGGAGCCAGATGA | 162 |
|  | Antisense: AAGATTGAAGCGAGAA |  |
| OR6 | Sense: CTCCGTGTTGTTTGG | 195 |
|  | Antisense: TTCGTGCTCTTATCGA |  |
| OR7 | Sense: CTCTTCATTCCCTGCTA | 107 |
|  | Antisense: TTTCTTTCCTCCTTTGT |  |
| OR8 | Sense: GGGAACGATGATTGA | 156 |
|  | Antisense: AATGGCCCACCAATA |  |
| Gustatory receptors | |  |
| GR1 | Sense: TTTGATTCCGTCCCTT | 153 |
|  | Antisense: CGTCGTCTACCGATTAG |  |
| GR2 | Sense: TCAAATGACCAATGCTGA | 154 |
|  | Antisense: TGATGTACTGCGGATGA |  |
| GR3 | Sense: ATCAAGCGTATGAAGGA | 170 |
|  | Antisense: AATGGCGTTGGAGTG |  |
| GR4 | Sense: AGTTCATTTCGTTGCT | 149 |
|  | Antisense: CTTTTCACTGATGCTTT |  |
| GR5 | Sense: AATGGACGAAACAAAC | 175 |
|  | Antisense: ATCATCCTCGCAAATA |  |
| GR6 | Sense: GGCTTTGAATCGTTTGC | 110 |
|  | Antisense: CTTGGCGTTTAATTTCG |  |
| GR7 | Sense: TCGCTTTCAAATGGTT  Antisense: GTCCTTTGCGAGATTG | 191 |
| GR8 | Sense: TGTAACGCATATTCATCAG | 102 |
|  | Antisense: GCAATTCAGCGAGGC |  |
| GR9 | Sense: GCCGTTCCAAGGGCATCT | 129 |
|  | Antisense: TCCGCAAACCTTCAGCACA |  |
| GR10 | Sense: ACAAGCTATTTAATAATTC | 147 |
|  | Antisense: AGTGGTGAGTGAAATAAATG |  |
| GR11 | Sense: TGAAACTGAGCGTATGT | 117 |
|  | Antisense: TAAGTGACCAGCGAAC |  |
| GR12 | Sense: TGGCAAGATGGTGAC | 151 |
|  | Antisense: GTACATTCGGCTCGT |  |
| Chemosensory proteins | |  |
| CSP1 | Sense: ATTGATAATCGTCCTG | 101 |
|  | Antisense: TCATTGCTATCAGACTT |  |
| Ionotropic receptors | |  |
| IR1 | Sense: TTAATGAACGCTACAAACG | 117 |
|  | Antisense: AGGGAGCAGTAAACAAATAGA |  |
| IR2 | Sense: GGCGGTGGTGGTAAT | 104 |
|  | Antisense: TCAGCAGGCAGTGGTT |  |
| IR3 | Sense: GTCAGCATCGGGAGCA | 173 |
|  | Antisense: CGAACCAAACGCACT |  |
| IR4 | Sense: ATTGTATTGCGGTGAAG | 199 |
|  | Antisense: AAATCCATCTAAGCCAGT |  |
| IR5 | Sense: TCTCGTCAACGGCAACC | 169 |
|  | Antisense: GGCGGAAAGTGTAAGGATG |  |
| IR6 | Sense: TTGCATCACGCCTCG | 200 |
|  | Antisense: ATCCCATTTCTTTGTCACTT |  |
| IR7 | Sense: TTTCTGCCGTAAGTCC | 192 |
|  | Antisense: ATCCCGATGATCCAAT |  |
| IR8 | Sense: TCCCAAACTCGGTCAA | 106 |
|  | Antisense: GCGTACCAAGGCATC |  |
| IR9 | Sense: AATTACGAGCGCAACC | 115 |
|  | Antisense: GTGGAAGACCGATTTGA |  |
| IR10 | Sense: TTCAACGCCCAAAGC | 168 |
|  | Antisense: TTGCCGAAAGGAAGC |  |
| IR11 | Sense: TATGACAAGTGGTGGAAA | 153 |
|  | Antisense: AATGATGGCTATAAGAACAG |  |
| IR12 | Sense: AAACTGGAGCCGTGAA | 119 |
|  | Antisense: TGGCGATGTAAAGTAGGA |  |
| IR13 | Sense: GCCATTGGCATGAGT | 116 |
|  | Antisense: GGCAATCCACCGTAC |  |
| IR14 | Sense: CGGCTTTATTTCGTCAA | 138 |
|  | Antisense: GGTAAGACAACGAGCACAA |  |
| IR15 | Sense: TTTCAAAGCCCTTCAT | 124 |
|  | Antisense: GCAGCGAGAACATAGAG |  |
| IR16 | Sense: CTGAAAGAGTCGGGAGA | 155 |
|  | Antisense: AGTAGACCGCCAATAAGA |  |
| Sensory neuron membrane proteins | |  |
| SNMP1 | Sense: CGTTGCTTCACCCAT | 138 |
|  | Antisense: GGAGTTCCCGTAGTCG |  |
| reference gene | |  |
| β-tubulin | Sense: GTTCCGTCGCAAAGC | 155 |
|  | Antisense: CTTCATCTTCACCCTCAAT |  |
