# Supplementary material for: Identification and expression pattern of chemosensory genes in the transcriptome of Propsilocerus akamusi
Source: PeerJ. 2020 Jul 21;8:e9584. doi: 10.7717/peerj.9584 (PMC7380273; doi:10.7717/peerj.9584)
Supplement: Supplemental Information 5 [file peerj-08-9584-s005.docx]

Table S2. The list and the nucleotide sequences of 34 OBPs of *P. akamusi* identified in present study.

| Unigene | Gene name | Accession number | ORF(bp) | Complete ORF | Blastx annotation | Score | e_value | Identity (%) |
| --- | --- | --- | --- | --- | --- | --- | --- | --- |
| Unigene5544_All | PaOBP1 | MN132992 | 438 | Yes | gi\|573006056\|gb\|AHF71056.1\|/5.38028e-07/odorant-binding protein 25 *[Lygus lineolaris]* | 61.23 | 5.7e-17 | 29.95 |
| Unigene2192_All | PaOBP2 | MN132993 | 429 | Yes | gi\|1000196531\|gb\|KXJ71014.1\|/8.02266e-21/hypothetical protein RP20_CCG021771 *[Aedes albopictus]* | 105.92 | 8.02e-21 | 43.59 |
| Unigene2198_All | PaOBP3 | MN132994 | 519 | Yes | gi\|573006056\|gb\|AHF71056.1\|/3.06836e-08/odorant-binding protein 25 *[Lygus lineolaris]* | 63.54 | 5.4e-07 | 29.95 |
| Unigene8580_All | PaOBP4 | MN132995 | 342 | Yes | gi\|668462257\|gb\|KFB49726.1\|/3.20749e-25/AGAP012321-PA-like protein *[Anopheles sinensis]* | 118.63 | 3.2e-25 | 45.6 |
| Unigene16967_All | PaOBP5 | MN132996 | 318 | No | gi\|668451132\|gb\|KFB40313.1\|/1.18954e-08/odorant binding protein (AGAP007289-PA) *[Anopheles sinensis]* | 63.54 | 3.5e-08 | 26.83 |
| Unigene4956_All | PaOBP6 | MN132997 | 348 | Yes | gi\|1000196531\|gb\|KXJ71014.1\|/5.14476e-18/hypothetical protein RP20_CCG021771 *[Aedes albopictus]* | 95.52 | 5.1e-18 | 40.71 |
| Unigene4999_All | PaOBP7 | MN132998 | 483 | Yes | gi\|1000210466\|gb\|KXJ80437.1\|/7.98036e-16/hypothetical protein RP20_CCG024996 *[Aedes albopictus]* | 87.42 | 3.7e-05 | 37.21 |
| Unigene19816_All | PaOBP8 | MN132999 | 393 | Yes | gi\|195108539\|ref\|XP_001998850.1\|/7.54395e-20/Odorant-binding protein 99a *[Drosophila mojavensis]* | 99.37 | 3.7e-19 | 38.64 |
| Unigene19940_All | PaOBP9 | MN133000 | 417 | No | gi\|157103281\|ref\|XP_001647907.1\|/7.82221e-22/AAEL000035-PA, partial *[Aedes aegypti]* | 108.61 | 7.8e-22 | 37.16 |
| Unigene19203_All | PaOBP10 | MN133001 | 339 | No | gi\|1000196531\|gb\|KXJ71014.1\|/1.14336e-14/hypothetical protein RP20_CCG021771 *[Aedes albopictus]* | 83.57 | 1.1e-14 | 32.2 |
| CL328.Contig3_All | PaOBP11 | MN133002 | 414 | Yes | gi\|668462264\|gb\|KFB49733.1\|/1.78462e-10/AGAP012318-PA-like protein *[Anopheles sinensis]* | 70.86 | 1.8e-10 | 35.87 |
| Unigene19633_All | PaOBP12 | MN133003 | 264 | No | gi\|340396202\|gb\|AEK32391.1\|/1.87767e-09/plus-C odorant-binding protein 2 *[Culex quinquefasciatus]* | 64.70 | 5.5e-09 | 35.96 |
| Unigene17339_All | PaOBP13 | MN133004 | 429 | Yes | gi\|568251030\|gb\|ETN60627.1\|/2.61652e-06/odorant binding protein *[Anopheles darlingi]* | 55.07 | 4.5e-06 | 37.5 |
| Unigene19933_All | PaOBP14 | MN133005 | 138 | No | gi\|568256504\|gb\|ETN65011.1\|/9.32467e-09/odorant binding protein *[Anopheles darlingi]* | 63.93 | 9.3e-09 | 46.27 |
| Unigene00667_All | PaOBP15 | MN133006 | 390 | Yes | gi\|668462257\|gb\|KFB49726.1\|AGAP012321-PA-like protein *[Anopheles sinensis]* | 100 | 1.90e-25 | 42.28 |
| Unigene02367_All | PaOBP16 | MN133007 | 927 | Yes | gi\|157129473\|ref\|XP_001661692.1\|odorant-binding protein 58c, putative *[Aedes aegypti]* | 68 | 1.67e-11 | 31.03 |
| Unigene03103_All | PaOBP17 | MN133008 | 414 | Yes | gi\|568256261\|gb\|ETN64831.1\|odorant binding protein *[Anopheles darlingi]* | 76 | 1.09e-15 | 32.35 |
| Unigene03115_All | PaOBP18 | MN133009 | 606 | Yes | gi\|157129473\|ref\|XP_001661692.1\|odorant-binding protein 58c, putative *[Aedes aegypti]* | 82 | 3.04e-17 | 31.21 |
| Unigene03420_All | PaOBP19 | MN133010 | 549 | Yes | gi\|170054451\|ref\|XP_001863135.1\|general odorant-binding protein 56d *[Culex quinquefasciatus]* | 83 | 2.56e-18 | 41.90 |
| Unigene03770_All | PaOBP20 | MN133011 | 303 | Yes | gi\|409107008\|pdb\|3VB1\|A\|Chain A, Crystal Structure Of *Anopholes Gambiae* odorant Binding Protein 20 In Open State | 104 | 2.14e-27 | 43.97 |
| Unigene04666_All | PaOBP21 | MN133012 | 429 | Yes | gi\|170063778\|ref\|XP_001867251.1\|odorant-binding protein 56e *[Culex quinquefasciatus]* | 78 | 1.05e-16 | 32.23 |
| Unigene05906_All | PaOBP22 | MN133013 | 750 | Yes | gi\|662036374\|gb\|AIE43918.1\|odorant-binding protein 29, partial *[Lutzomyia longipalpis]* | 173 | 6.39e-51 | 42.11 |
| Unigene06716_All | PaOBP23 | MN133014 | 642 | Yes | gi\|157129495\|ref\|XP_001661703.1\|odorant-binding protein 50d, putative *[Aedes aegypti]* | 60 | 1.18e-09 | 31.33 |
| Unigene06762_All | PaOBP24 | MN133015 | 645 | Yes | gi\|568251290\|gb\|ETN60853.1\|odorant binding protein *[Anopheles darlingi]* | 80 | 9.32e-17 | 34.95 |
| Unigene07342_All | PaOBP25 | MN133016 | 465 | Yes | gi\|751438043\|ref\|XP_011176687.1\|PREDICTED: general odorant-binding protein lush isoform X1 *[Bactrocera cucurbitae]* | 139 | 1.22e-39 | 46.15 |
| Unigene07992_All | PaOBP26 | MN133017 | 429 | Yes | gi\|157123243\|ref\|XP_001660077.1\|odorant binding protein *[Aedes aegypti]* | 231 | 5.16e-76 | 75.00 |
| Unigene08354_All | PaOBP27 | MN133018 | 573 | Yes | gi\|311707030\|gb\|ADQ01710.1\|odorant binding protein 24 *[Anopheles funestus]* | 72 | 6.25e-14 | 27.08 |
| Unigene09189_All | PaOBP28 | MN133019 | 561 | Yes | gi\|157129477\|ref\|XP_001661694.1\|odorant-binding protein 47b, putative *[Aedes aegypti]* | 98 | 4.26e-23 | 33.33 |
| Unigene09193_All | PaOBP29 | MN133020 | 408 | Yes | gi\|157111228\|ref\|XP_001651445.1\|odorant-binding protein 99c, putative *[Aedes aegypti]* | 157 | 2.22e-47 | 56.52 |
| Unigene09360_All | PaOBP30 | MN133021 | 453 | Yes | gi\|311707028\|gb\|ADQ01709.1\|odorant binding protein 20 *[Anopheles funestus]* | 145 | 1.75e-42 | 53.28 |
| Unigene10306_All | PaOBP31 | MN133022 | 579 | Yes | gi\|340396202\|gb\|AEK32391.1\|plus-C odorant-binding protein 2 *[Culex quinquefasciatus]* | 90 | 4.29e-20 | 27.66 |
| Unigene10384_All | PaOBP32 | MN133023 | 426 | Yes | gi\|471178529\|gb\|AGI04314.1\|obp14 *[Aedes albopictus]* | 90 | 3.35e-21 | 38.10 |
| Unigene10557_All | PaOBP33 | MN133024 | 453 | Yes | gi\|668462262\|gb\|KFB49731.1\|odorant binding protein 24 *[Anopheles sinensis]* | 62 | 1.17e-10 | 25.20 |
| Unigene11219_All | PaOBP34 | MN133025 | 426 | Yes | gi\|31239625\|ref\|XP_320226.1\|AGAP012318-PA *[Anopheles gambiae str. PEST]* | 74 | 2.16e-15 | 35.16 |
