# Supplementary material for: Identification and expression pattern of chemosensory genes in the transcriptome of Propsilocerus akamusi
Source: PeerJ. 2020 Jul 21;8:e9584. doi: 10.7717/peerj.9584 (PMC7380273; doi:10.7717/peerj.9584)
Supplement: Supplemental Information 6 [file peerj-08-9584-s006.docx]

Table S3. The list and the nucleotide sequences of 17 ORs of *P. akamusi* identified in present study.

| Unigene | Gene name | Accession number | ORF(bp) | Complete ORF | Blastx annotation | Score | e_value | Identity (%) |
| --- | --- | --- | --- | --- | --- | --- | --- | --- |
| Unigene1186_All | PaOR1 | MN133026 | 1383 | Yes | gi\|749755907\|ref\|XP_011140452.1\|/2.42783e-12/PREDICTED: odorant receptor 46a, isoform A-like *[Harpegnathos saltator]* | 78.9518 | 2.43e-12 | 21.64 |
| CL2639.Contig2_All | PaOR2 | MN133027 | 1326 | Yes | gi\|31207639\|ref\|XP_312786.1\|/4.75981e-148/AGAP003098-PA *[Anopheles gambiae str. PEST]* | 340.502 | 4.76e-148 | 69.77 |
| Unigene3449_All | PaOR3 | MN133028 | 1395 | Yes | gi\|685453860\|gb\|AIO10777.1\|/0/odorant receptor co-receptor *[Anopheles funestus]* | 765.377 | 0 | 80.17 |
| Unigene20280_All | PaOR4 | MN133029 | 813 | Yes | gi\|170036073\|ref\|XP_001845890.1\|/5.68872e-14/Odorant receptor 92a *[Culex quinquefasciatus]* | 81.2629 | 5.69e-14 | 40.4 |
| CL1665.Contig2_All | PaOR5 | MN133030 | 1671 | Yes | gi\|906461341\|gb\|KNC23846.1\|/9.34228e-19/putative odorant receptor 94b, partial *[Lucilia cuprina]* | 100.138 | 9.34e-19 | 21.76 |
| Unigene18711_All | PaOR6 | MN133031 | 312 | No | gi\|31237083\|ref\|XP_319538.1\|/5.353e-20/AGAP003310-PA *[Anopheles gambiae str. PEST]* | 101.293 | 5.35e-20 | 38.79 |
| Unigene14540_All | PaOR7 | MN133032 | 1206 | Yes | gi\|751780083\|ref\|XP_011199152.1\|/7.12439e-09/PREDICTED: LOW QUALITY PROTEIN: odorant receptor 67c-like *[Bactrocera dorsalis]* | 64.3142 | 7.12e-09 | 39.73 |
| Unigene00454_All | PaOR8 | MN133033 | 966 | Yes | gi\|170060086\|ref\|XP_001865647.1\|Odorant receptor 56a *[Culex quinquefasciatus]* | 261 | 3.53E-81 | 42.17 |
| Unigene02382_All | PaOR9 | MN133034 | 720 | Yes | gi\|666916209\|gb\|AIG51899.1\|odorant receptor *[Helicoverpa armigera]* | 106 | 1.69E-24 | 29.88 |
| Unigene03213_All | PaOR10 | MN133035 | 1164 | Yes | gi\|195166334\|ref\|XP_002023990.1\|GL27118 *[Drosophila persimilis]* | 96 | 4.67E-20 | 23.62 |
| Unigene03624_All | PaOR11 | MN133036 | 1080 | Yes | gi\|380011604\|ref\|XP_003689890.1\|PREDICTED: putative odorant receptor 94b-like *[Apis florea]* | 77 | 7.18E-14 | 27.09 |
| Unigene05048_All | PaOR12 | MN133037 | 1158 | Yes | gi\|195054076\|ref\|XP_001993952.1\|GH22367 *[Drosophila grimshawi]* | 129 | 3.49E-31 | 26.88 |
| Unigene05457_All | PaOR13 | MN133038 | 1209 | Yes | gi\|195159423\|ref\|XP_002020578.1\|GL15322 *[Drosophila persimilis]* | 197 | 5.40E-56 | 31.25 |
| Unigene05827_All | PaOR14 | MN133039 | 1350 | Yes | gi\|170039362\|ref\|XP_001847506.1\|odorant response protein ODR-4 *[Culex quinquefasciatus]* | 258 | 6.27E-78 | 34.96 |
| Unigene08549_All | PaOR15 | MN133040 | 1146 | Yes | gi\|24664442\|ref\|NP_524078.2\|odorant receptor 71a, isoform B *[Drosophila melanogaster]* | 129 | 1.89E-31 | 26.52 |
| Unigene09465_All | PaOR16 | MN133041 | 1035 | Yes | gi\|170047391\|ref\|XP_001851206.1\|olfactory receptor *[Culex quinquefasciatus]* | 178 | 5.15E-49 | 31.94 |
| Unigene11815_All | PaOR17 | MN133042 | 1143 | Yes | gi\|194910664\|ref\|XP_001982203.1\|GG11175 *[Drosophila erecta]* | 118 | 1.69E-27 | 23.98 |
