# Supplementary material for: Identification and expression pattern of chemosensory genes in the transcriptome of Propsilocerus akamusi
Source: PeerJ. 2020 Jul 21;8:e9584. doi: 10.7717/peerj.9584 (PMC7380273; doi:10.7717/peerj.9584)
Supplement: Supplemental Information 7 [file peerj-08-9584-s007.docx]

Table S4. The list and the nucleotide sequences of 32 GRs of *P. akamusi* identified in present study.

| Unigene | Gene name | Accession number | ORF(bp) | Complete ORF | Blastx annotation | Score | e_value | Identity (%) |
| --- | --- | --- | --- | --- | --- | --- | --- | --- |
| CL907.Contig2_All | PaGR1 | MN132938 | 1839 | Yes | gi\|969474873\|ref\|XP_015038251.1\|/1.47932e-140/uncharacterized protein Dpse_GA16009, isoform B [Drosophila pseudoobscura pseudoobscura] | 362.84 | 1.48e-140 | 54.14 |
| Unigene4874_All | PaGR2 | MN132939 | 432 | Yes | gi\|906458862\|gb\|KNC22003.1\|/2.57152e-20/putative gustatory receptor 59f [Lucilia cuprina] | 103.99 | 2.57e-20 | 37.72 |
| Unigene4494_All | PaGR3 | MN132940 | 597 | No | gi\|195481628\|ref\|XP_002101716.1\|/3.4589e-11/uncharacterized protein Dyak_GE17782 [Drosophila yakuba] | 73.94 | 3.46e-11 | 24.55 |
| Unigene13846_All | PaGR4 | MN132941 | 294 | Yes | gi\|568252095\|gb\|ETN61475.1\|/7.07845e-21/allatostatin 2 [Anopheles darlingi] | 107.07 | 7.08e-21 | 48.36 |
| Unigene7969_All | PaGR5 | MN132942 | 333 | No | gi\|568249794\|gb\|ETN59575.1\|/1.0241e-10/gustatory receptor [Anopheles darlingi] | 70.48 | 1.02e-10 | 30.97 |
| Unigene7981_All | PaGR6 | MN132943 | 258 | No | gi\|170062194\|ref\|XP_001866562.1\|/2.0997e-08/conserved hypothetical protein [Culex quinquefasciatus] | 62.77 | 2.10e-08 | 27.97 |
| Unigene14322_All | PaGR7 | MN132944 | 474 | No | gi\|906469696\|gb\|KNC30394.1\|/1.59922e-43/putative gustatory receptor 64f [Lucilia cuprina] | 180.64 | 1.60e-43 | 41.41 |
| Unigene11996_All | PaGR8 | MN132945 | 1575 | Yes | gi\|568255233\|gb\|ETN64058.1\|/9.34363e-33/gustatory receptor [Anopheles darlingi] | 143.67 | 9.34e-33 | 63.96 |
| Unigene15118_All | PaGR9 | MN132946 | 390 | No | gi\|194748643\|ref\|XP_001956754.1\|/1.51601e-41/uncharacterized protein Dana_GF10085 [Drosophila ananassae] | 174.10 | 1.52e-41 | 46.41 |
| Unigene15291_All | PaGR10 | MN132947 | 219 | No | gi\|951550797\|ref\|XP_014475219.1\|/8.85749e-07/PREDICTED: gustatory receptor for sugar taste 64f-like isoform X7 [Dinoponera quadriceps] | 57.38 | 8.86e-07 | 35.29 |
| Unigene19587_All | PaGR11 | MN132948 | 1209 | Yes | gi\|194759770\|ref\|XP_001962120.1\|/5.07234e-10/uncharacterized protein Dana_GF14599 [Drosophila ananassae] | 68.17 | 5.07e-10 | 37.89 |
| Unigene2820_All | PaGR12 | MN132949 | 1293 | Yes | gi\|929365084\|ref\|XP_014092168.1\|/2.76667e-53/PREDICTED: LOW QUALITY PROTEIN: gustatory receptor for sugar taste 64b-like [Bactrocera oleae] | 214.54 | 2.77e-53 | 38.1 |
| Unigene4002_All | PaGR13 | MN132950 | 558 | No | gi\|170062427\|ref\|XP_001866663.1\|/1.13703e-69/gustatory receptor for trehalose [Culex quinquefasciatus] | 269.24 | 1.14e-69 | 54.62 |
| CL1143.Contig1_All | PaGR14 | MN132951 | 1365 | Yes | gi\|347969553\|ref\|XP_307764.3\|/7.82426e-63/AGAP003256-PA [Anopheles gambiae str. PEST] | 247.28 | 7.82e-63 | 33.25 |
| Unigene2676_All | PaGR15 | MN132952 | 792 | No | gi\|765332942\|ref\|XP_001647880.2\|/3.85121e-47/AAEL000060-PA, partial [Aedes aegypti] | 193.36 | 3.85e-47 | 36.63 |
| Unigene8790_All | PaGR16 | MN132953 | 177 | No | gi\|985403218\|ref\|XP_015369415.1\|/6.9512e-12/PREDICTED: gustatory receptor for sugar taste 64f-like isoform X2 [Diuraphis noxia] | 74.33 | 6.95e-12 | 35.58 |
| Unigene10519_All | PaGR17 | MN132954 | 414 | No | gi\|1000205604\|gb\|KXJ76746.1\|/7.14688e-16/hypothetical protein RP20_CCG009050 [Aedes albopictus] | 87.81 | 7.15e-16 | 31.18 |
| Unigene12040_All | PaGR18 | MN132955 | 1656 | Yes | gi\|668455345\|gb\|KFB43629.1\|/1.0269e-07/AGAP001117-PD-like protein [Anopheles sinensis] | 60.46 | 1.03e-07 | 29.93 |
| Unigene13289_All | PaGR19 | MN132956 | 342 | Yes | gi\|668455345\|gb\|KFB43629.1\|/2.57671e-26/AGAP001117-PD-like protein [Anopheles sinensis] | 125.95 | 2.58e-26 | 26.34 |
| Unigene14624_All | PaGR20 | MN132957 | 3798 | Yes | gi\|906469696\|gb\|KNC30394.1\|/1.01314e-18/putative gustatory receptor 64f [Lucilia cuprina] | 97.06 | 1.01e-18 | 53.66 |
| Unigene15474_All | PaGR21 | MN132958 | 93 | No | gi\|1000206675\|gb\|KXJ77535.1\|/7.59654e-06/hypothetical protein RP20_CCG007326 [Aedes albopictus] | 54.30 | 7.60e-06 | 33.33 |
| Unigene15779_All | PaGR22 | MN132959 | 210 | No | gi\|158284679\|ref\|XP_307760.4\|/1.31386e-18/AGAP003253-PA [Anopheles gambiae str. PEST] | 96.67 | 1.31e-18 | 41.75 |
| Unigene15784_All | PaGR23 | MN132960 | 1191 | Yes | gi\|170062427\|ref\|XP_001866663.1\|/4.27929e-38/gustatory receptor for trehalose [Culex quinquefasciatus] | 161.38 | 4.28e-38 | 62.16 |
| Unigene17441_All | PaGR24 | MN132961 | 1404 | Yes | gi\|170041909\|ref\|XP_001848689.1\|/2.42193e-20/gustatory receptor 24 [Culex quinquefasciatus] | 102.45 | 2.42e-20 | 60.49 |
| Unigene00008_All | PaGR25 | MN132962 | 1527 | Yes | gi\|158284679\|ref\|XP_307760.4\|AGAP003253-PA *[Anopheles gambiae str. PEST]* | 112 | 2.44e-30 | 55.88 |
| Unigene00539_All | PaGR26 | MN132963 | 1044 | Yes | gi\|642929128\|ref\|XP_008195702.1\|PREDICTED: putative gustatory receptor 28b *[Tribolium castaneum]* | 203 | 9.37e-57 | 39.60 |
| Unigene01689_All | PaGR27 | MN132964 | 1236 | Yes | gi\|557783627\|ref\|XP_005191421.1\|PREDICTED: putative gustatory receptor 2a *[Musca domestica]* | 68 | 1.04e-10 | 33.07 |
| Unigene02453_All | PaGR28 | MN132965 | 2079 | Yes | gi\|642936692\|ref\|XP_008198539.1\|PREDICTED: glutamate receptor ionotropic, delta-2 *[Tribolium castaneum]* | 100 | 2.30e-21 | 25.78 |
| Unigene04269_All | PaGR29 | MN132966 | 2877 | Yes | gi\|568252261\|gb\|ETN61614.1\|Ionotropic receptor 21a *[Anopheles darlingi]* | 538 | 2.63e-180 | 44.81 |
| Unigene04990_All | PaGR30 | MN132967 | 1893 | Yes | gi\|499009072\|ref\|XP_004536475.1\|PREDICTED: putative gustatory receptor 2a-like *[Ceratitis capitata]* | 862 | 0 | 53.27 |
| Unigene10785_All | PaGR31 | MN132968 | 258 | Yes | gi\|498982126\|ref\|XP_004529782.1\|PREDICTED: putative gustatory receptor 66a-like *[Ceratitis capitata]* | 133 | 1.90e-31 | 29.36 |
| Unigene11260_All | PaGR32 | MN132969 | 1395 | Yes | gi\|157128603\|ref\|XP_001655150.1\|Gustatory receptor 21a, putative *[Aedes aegypti]* | 48 | 8.19e-06 | 42.37 |
