# Supplementary material for: Identification and expression pattern of chemosensory genes in the transcriptome of Propsilocerus akamusi
Source: PeerJ. 2020 Jul 21;8:e9584. doi: 10.7717/peerj.9584 (PMC7380273; doi:10.7717/peerj.9584)
Supplement: Supplemental Information 8 [file peerj-08-9584-s008.docx]

Table S5. The list and the nucleotide sequences of 22 IRs of *P. akamusi* identified in present study.

| Unigene | Gene name | Accession number | ORF(bp) | Complete ORF | Blastx annotation | Score | e_value | Identity (%) |
| --- | --- | --- | --- | --- | --- | --- | --- | --- |
| Unigene2499_All | PaIR1 | MN132970 | 2652 | Yes | gi\|170046685\|ref\|XP_001850884.1\|/0/conserved hypothetical protein [Culex quinquefasciatus] | 942.57 | 0.00e+00 | 52.06 |
| CL1432.Contig2_All | PaIR2 | MN132971 | 1581 | Yes | gi\|170056173\|ref\|XP_001863912.1\|/2.92459e-80/conserved hypothetical protein [Culex quinquefasciatus] | 305.06 | 2.92e-80 | 44.5 |
| Unigene5430_All | PaIR3 | MN132972 | 1617 | Yes | gi\|1000201333\|gb\|KXJ73839.1\|/7.17804e-169/hypothetical protein RP20_CCG014911 [Aedes albopictus] | 599.74 | 7.18e-169 | 56.83 |
| Unigene19944_All | PaIR4 | MN132973 | 1863 | Yes | gi\|157106493\|ref\|XP_001649349.1\|/3.06932e-75/AAEL014690-PA [Aedes aegypti] | 288.50 | 3.07e-75 | 31.38 |
| CL2030.Contig2_All | PaIR5 | MN132974 | 468 | Yes | gi\|668464906\|gb\|KFB52144.1\|/0/AGAP000803-PA-like protein [Anopheles sinensis] | 1308.51 | 0.00e+00 | 69.75 |
| CL2030.Contig3_All | PaIR6 | MN132975 | 2805 | Yes | gi\|568251481\|gb\|ETN61009.1\|/3.89825e-60/glutamate receptor, ionotropic kainate 1, 2, 3 (glur5, glur6, glur7) [Anopheles darlingi] | 234.96 | 3.90e-60 | 74.34 |
| Unigene6589_All | PaIR7 | MN132976 | 450 | Yes | gi\|347968576\|ref\|XP_312117.5\|/0/AGAP002797-PA [Anopheles gambiae str. PEST] | 1025.39 | 0.00e+00 | 58.58 |
| Unigene9808_All | PaIR8 | MN132977 | 1464 | Yes | gi\|170056615\|ref\|XP_001864109.1\|/6.80847e-23/ionotropic glutamate receptor [Culex quinquefasciatus] | 110.92 | 6.81e-23 | 54 |
| Unigene6551_All | PaIR9 | MN132978 | 333 | Yes | gi\|668464906\|gb\|KFB52144.1\|/0/AGAP000803-PA-like protein [Anopheles sinensis] | 913.29 | 0.00e+00 | 51.45 |
| CL639.Contig2_All | PaIR10 | MN132979 | 411 | Yes | gi\|939239369\|ref\|XP_014262401.1\|/1.49839e-151/PREDICTED: glutamate receptor ionotropic, kainate 2 [Cimex lectularius] | 542.35 | 1.50e-151 | 85.13 |
| Unigene13641_All | PaIR11 | MN132980 | 2622 | Yes | gi\|939239369\|ref\|XP_014262401.1\|/1.11948e-49/PREDICTED: glutamate receptor ionotropic, kainate 2 [Cimex lectularius] | 199.90 | 1.12e-49 | 70.59 |
| Unigene4339_All | PaIR12 | MN132981 | 267 | No | gi\|1000204182\|gb\|KXJ75762.1\|/1.34422e-15/hypothetical protein RP20_CCG011119 [Aedes albopictus] | 86.66 | 1.34e-15 | 40.74 |
| Unigene10587_All | PaIR13 | MN132982 | 5910 | Yes | gi\|668454898\|gb\|KFB43279.1\|/2.82004e-45/AGAP005527-PA-like protein [Anopheles sinensis] | 185.27 | 2.82e-45 | 73.04 |
| Unigene11312_All | PaIR14 | MN132983 | 276 | No | gi\|568258100\|gb\|ETN66290.1\|/1.20107e-11/hypothetical protein AND_001947 [Anopheles darlingi] | 73.56 | 1.20e-11 | 38.65 |
| Unigene13498_All | PaIR15 | MN132984 | 2895 | Yes | gi\|939239369\|ref\|XP_014262401.1\|/2.07964e-40/PREDICTED: glutamate receptor ionotropic, kainate 2 [Cimex lectularius] | 169.09 | 2.08e-40 | 94.25 |
| Unigene13542_All | PaIR16 | MN132985 | 2865 | Yes | gi\|805797811\|ref\|XP_012143663.1\|/1.88267e-55/PREDICTED: glutamate receptor 1-like [Megachile rotundata] | 221.09 | 1.88e-55 | 65.88 |
| Unigene1105_All | PaIR17 | MN132986 | 2847 | Yes | gi\|668464908\|gb\|KFB52146.1\|/0/AGAP002797-PA-like protein [Anopheles sinensis] | 928.32 | 0.00e+00 | 51.6 |
| Unigene3958_All | PaIR18 | MN132987 | 2817 | Yes | gi\|1000196554\|gb\|KXJ71028.1\|/0/hypothetical protein RP20_CCG021738 [Aedes albopictus] | 1511.89 | 0.00e+00 | 79.66 |
| CL2030.Contig1_All | PaIR19 | MN132988 | 393 | No | gi\|170068371\|ref\|XP_001868841.1\|/1.94388e-32/glutamate receptor [Culex quinquefasciatus] | 144.82 | 1.94e-32 | 55.41 |
| CL1105.Contig2_All | PaIR20 | MN132989 | 462 | Yes | gi\|668451008\|gb\|KFB40214.1\|/0/glutamate receptor, ionotropic, n-methyl d-aspartate epsilon (nmda epsilon) [Anopheles sinensis] | 1705.26 | 0.00e+00 | 83.81 |
| Unigene13631_All | PaIR21 | MN132990 | 2751 | Yes | gi\|662198318\|ref\|XP_008472277.1\|/1.05442e-92/PREDICTED: glutamate receptor ionotropic, kainate 2 [Diaphorina citri] | 345.13 | 1.05e-92 | 85.64 |
| Unigene12578_All | PaIR22 | MN132991 | 318 | Yes | gi\|357615043\|gb\|EHJ69440.1\|/4.58235e-24/hypothetical protein KGM_00270 [Danaus plexippus] | 114.78 | 4.58e-24 | 51.16 |
