# Supplementary material for: Identification and expression pattern of chemosensory genes in the transcriptome of Propsilocerus akamusi
Source: PeerJ. 2020 Jul 21;8:e9584. doi: 10.7717/peerj.9584 (PMC7380273; doi:10.7717/peerj.9584)
Supplement: Supplemental Information 9 [file peerj-08-9584-s009.docx]

Table S6. The list and the nucleotide sequences of 6 CSPs and 3 SNMP genes of *P. akamusi* identified in present study.

| Unigene | Gene name | Accession number | ORF(bp) | Complete ORF | Blastx annotation | Score | e_value | Identity (%) |
| --- | --- | --- | --- | --- | --- | --- | --- | --- |
| Unigene4958_All | PaCSP1 | MN132932 | 156 | Yes | gi\|281426843\|emb\|CBA11328.1\|/5.80983e-36/chemosensory protein 2 *[Glossina morsitans morsitans]* | 155.99 | 5.81e-36 | 55.74 |
| Unigene 07395_All | PaCSP2 | MN132933 | 285 | Yes | MG544171\|chemosensory protein 2 (CSP2)[ Bradysia odoriphaga] | 64.1 | 3e-06 | 79.31 |
| Unigene18310_All | PaCSP3 | MN132934 | 369 | No | gi\|925175954\|gb\|ALC79596.1\|/9.73377e-38/chemosensory protein 9 *[Grapholita molesta]* | 160.23 | 9.73e-38 | 57.72 |
| Unigene3999_All | PaCSP4 | MN132935 | 378 | No | gi\|215254082\|gb\|ACJ64053.1\|/5.31771e-68/putative chemosensory protein CSP6 *[Nilaparvata lugens]* | 260.77 | 5.32e-68 | 100 |
| Unigene3472_All | PaCSP5 | MN132936 | 132 | No | gi\|306850727\|gb\|ADN06871.1\|/2.50874e-38/chemosensory protein *[Nilaparvata lugens]* | 162.16 | 2.51e-38 | 100 |
| Unigene18891_All | PaCSP6 | MN132937 | 147 | No | gi\|554894795\|gb\|AGZ04929.1\|/8.50239e-26/chemosensory protein 1 *[Laodelphax striatella]* | 120.55 | 8.50e-26 | 88.71 |
| Unigene17684_All | PaSNMP1 | MN133043 | 1629 | Yes | gi\|340396196\|gb\|AEK32388.1\|/5.07906e-143/sensory neuron membrane protein 1c *[Culex quinquefasciatus]* | 513.457 | 5.08e-143 | 49.69 |
| Unigene 05708_All | PaSNMP2 | MN133044 | 1479 | Yes | gi\|332321832\|sp\|B0X4H5.2\|SNMP1_CULQU\|RecName: Full=Sensory neuron membrane protein 1 *[Culex quinquefasciatus]* | 430 | 9.31e-143 | 41.05 |
| Unigene 06894_All | PaSNMP3 | MN133045 | 1713 | Yes | gi\|374253753\|sp\|Q7Q6R1.5\|SNMP2_ANOGA\|RecName: Full=Sensory neuron membrane protein 2 *[Anopheles gambiae]* | 593 | 0 | 49.47 |
