# Supplementary figures and images for: Identification and expression pattern of chemosensory genes in the transcriptome of Propsilocerus akamusi
Source: PeerJ. 2020 Jul 21;8:e9584. doi: 10.7717/peerj.9584 (PMC7380273; doi:10.7717/peerj.9584)

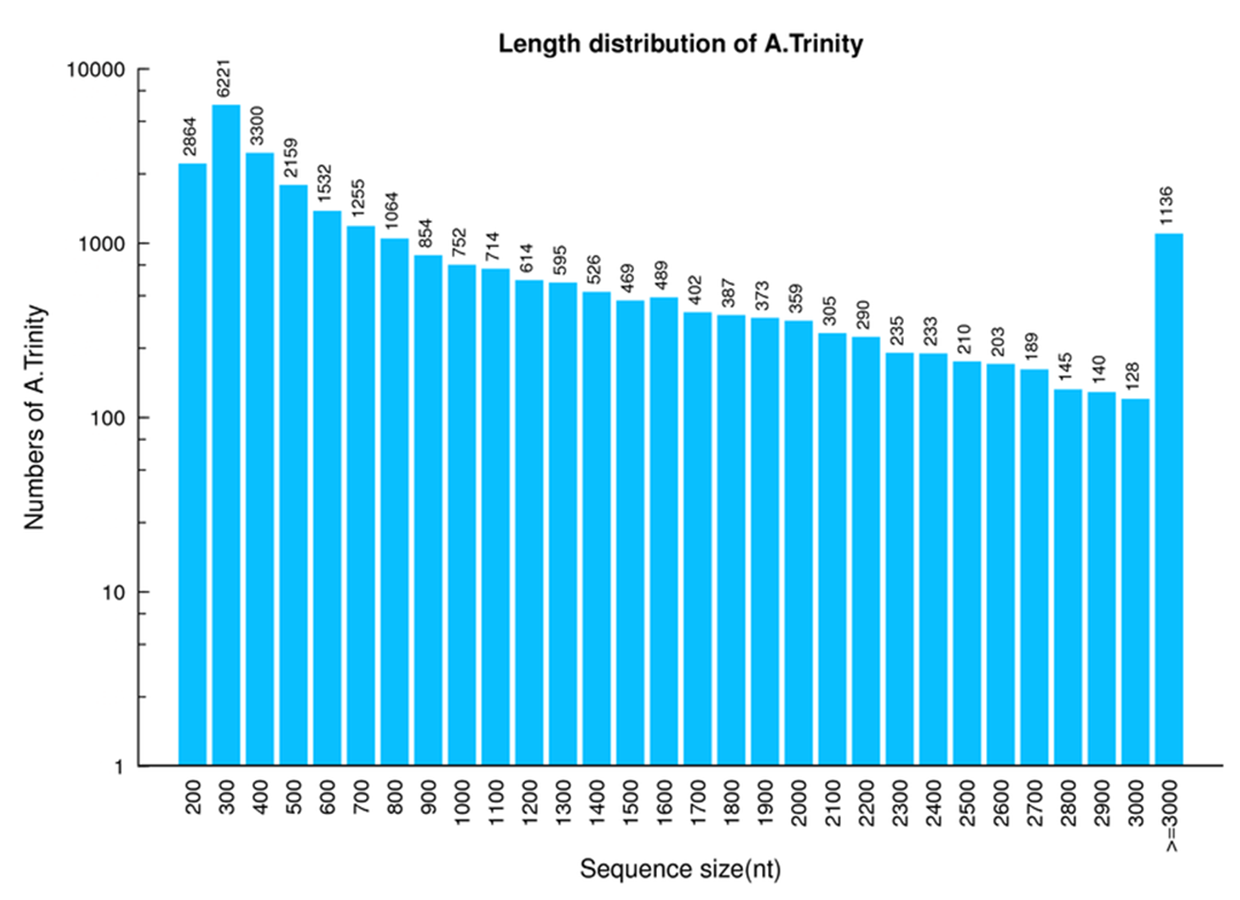

Supplement: Supplemental Information 10 — The x-axis represents the length of transcripts. The y-axis represents the number of transcripts. [file peerj-08-9584-s010.png]

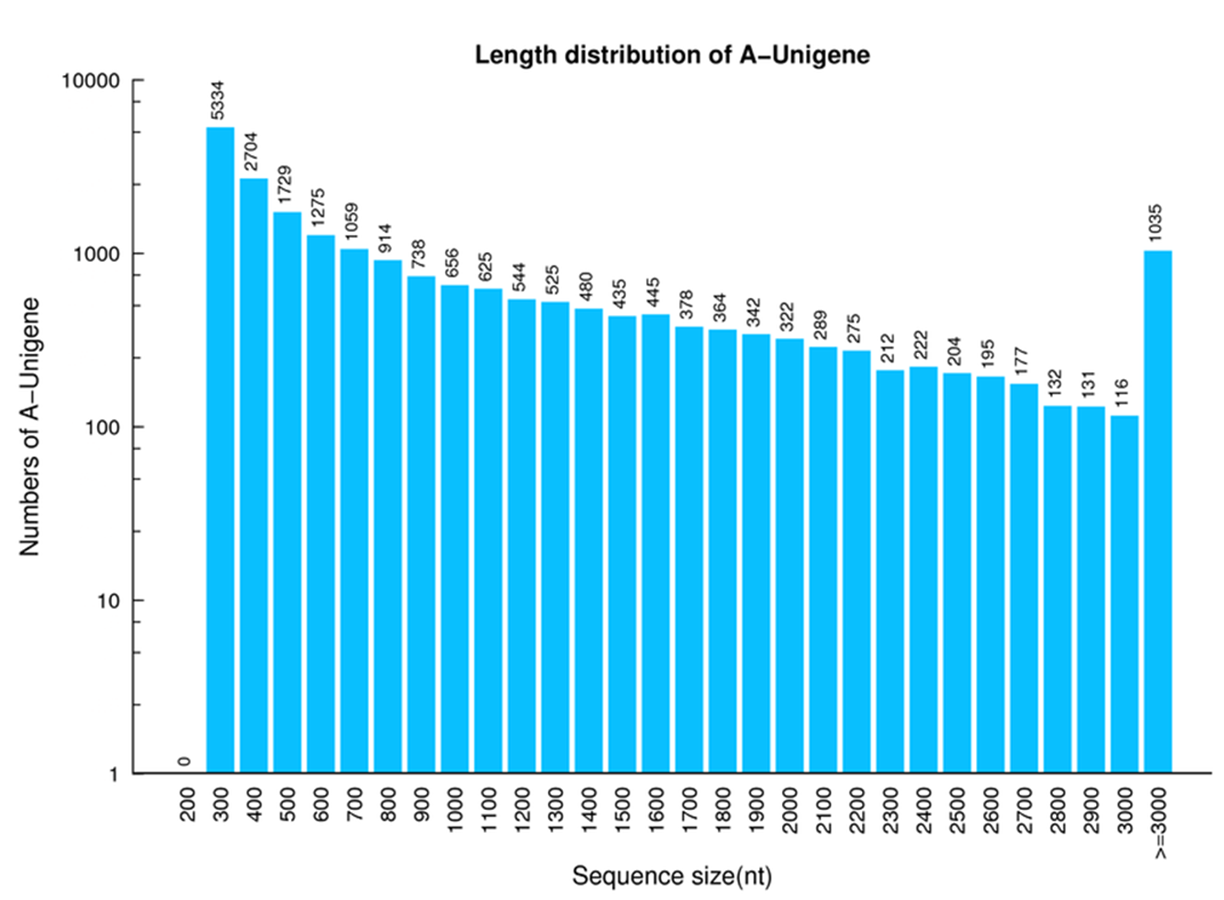

Supplement: Supplemental Information 11 — The x-axis represents the length of Unigenes. The y-axis represents the number of Unigenes. [file peerj-08-9584-s011.png]

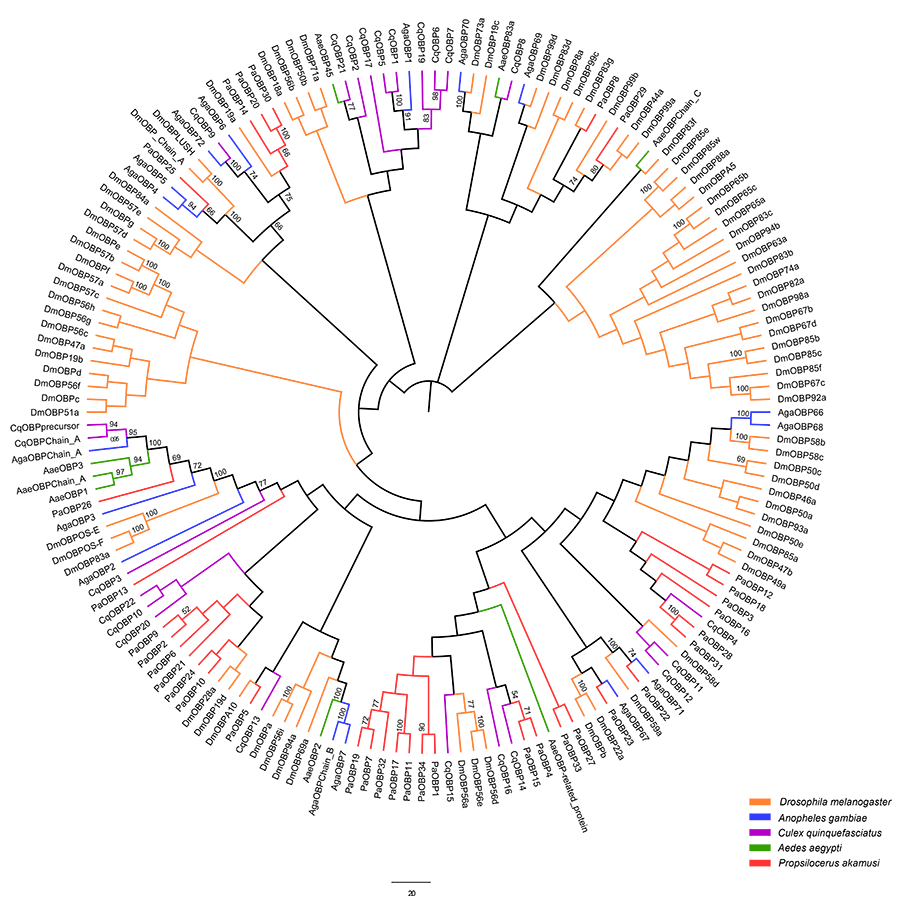

Supplement: Supplemental Information 12 — A. aegypti, A. gambiae, C. quinquefasciatus and P. akamusi. Bootstrap values were calculated with 1,000 replicates and the values < 50% are shown on the branches. Yellow: D. melanogaster; Blue: A. gambiae; Purple: C. quinquefasciatus; Green: A. aegypti; Red: P. akamusi. [file peerj-08-9584-s012.png]

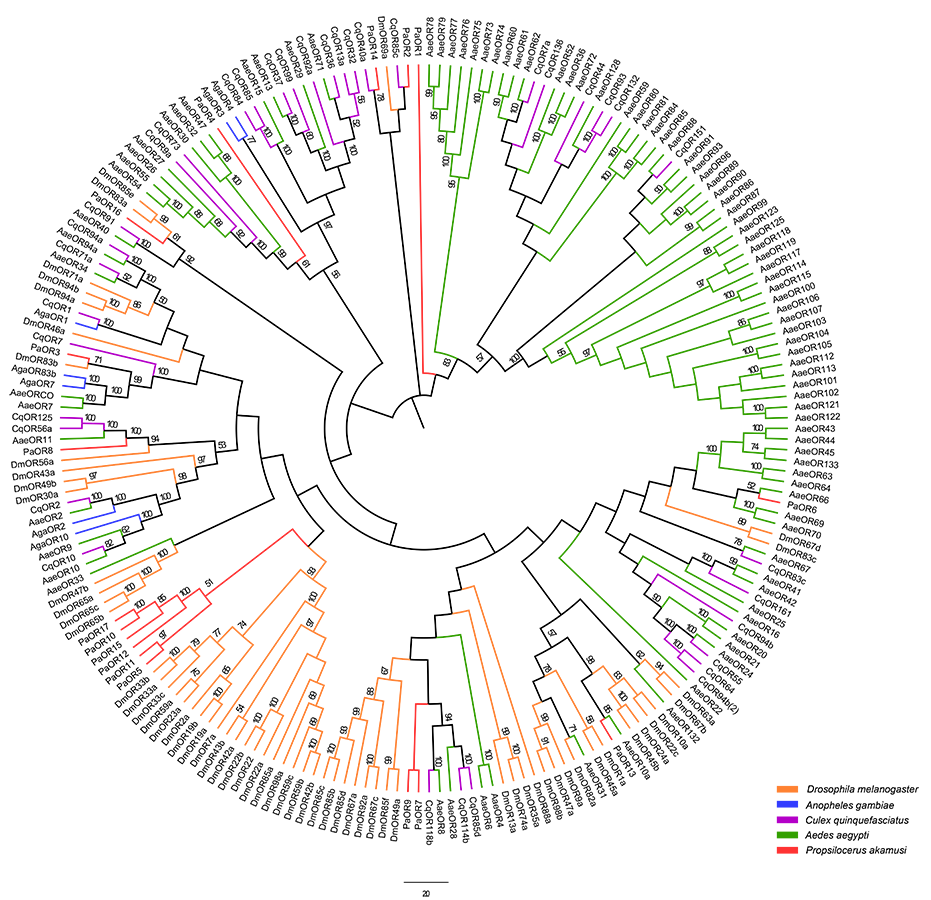

Supplement: Supplemental Information 13 — A. aegypti, A. gambiae, C. quinquefasciatus and P. akamusi. Bootstrap values were calculated with 1,000 replicates and the values < 50% are shown on the branches. Yellow: D. melanogaster; Blue: A. gambiae; Purple: C. quinquefasciatus; Green: A. aegypti; Red: P. akamusi. [file peerj-08-9584-s013.png]

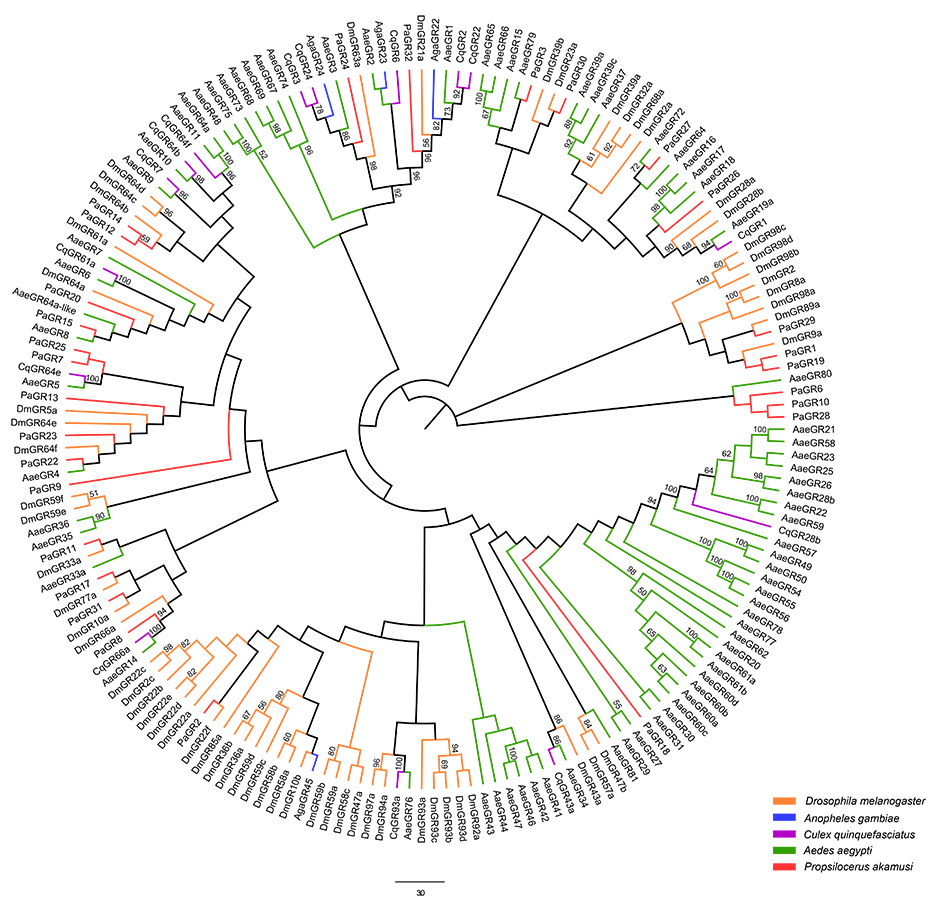

Supplement: Supplemental Information 14 — A. aegypti, A. gambiae, C. quinquefasciatus and P. akamusi. Bootstrap values were calculated with 1000 replicates and the values < 50% are shown on the branches. Yellow: D. melanogaster; Blue: A. gambiae; Purple: C. quinquefasciatus; Green: A. aegypti; Red: P. akamusi. [file peerj-08-9584-s014.png]

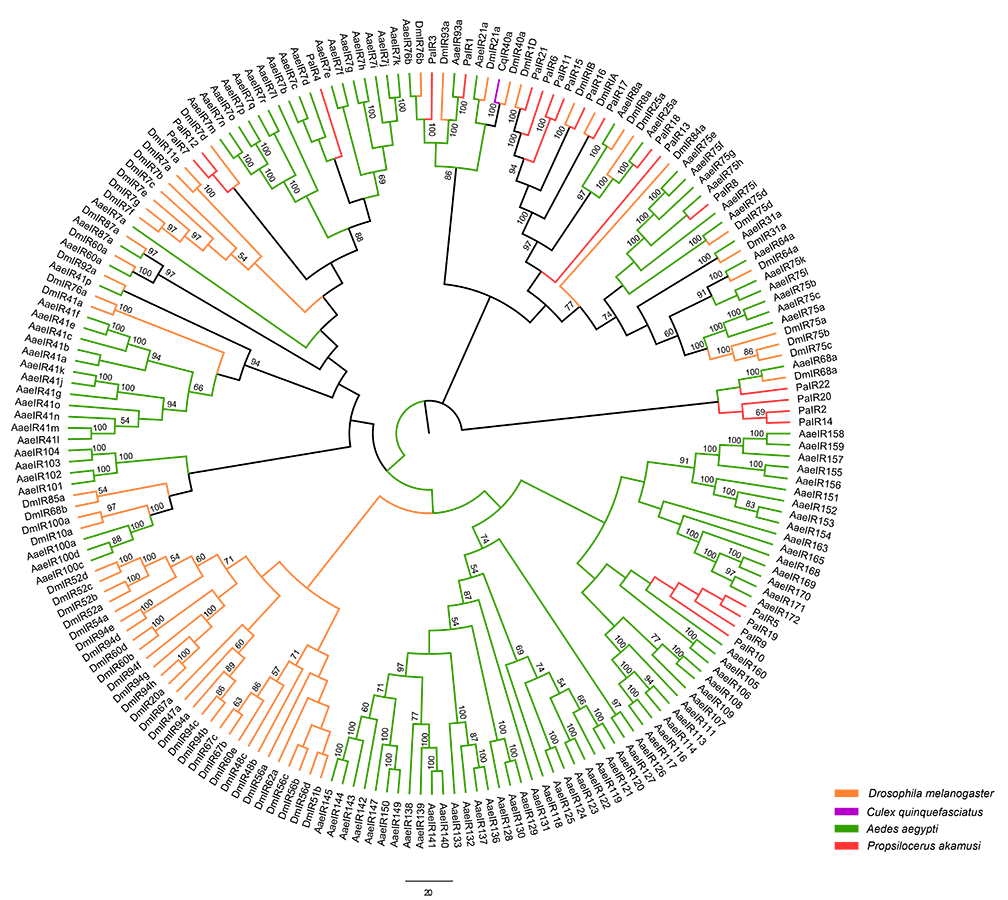

Supplement: Supplemental Information 15 — A. aegypti, A. gambiae, and P. akamusi. Bootstrap values were calculated with 1,000 replicates and the values ¿50% are shown on the branches. Yellow: D. melanogaster; Blue: A. gambiae; Green: A. aegypti; Red: P. akamusi. [file peerj-08-9584-s015.png]

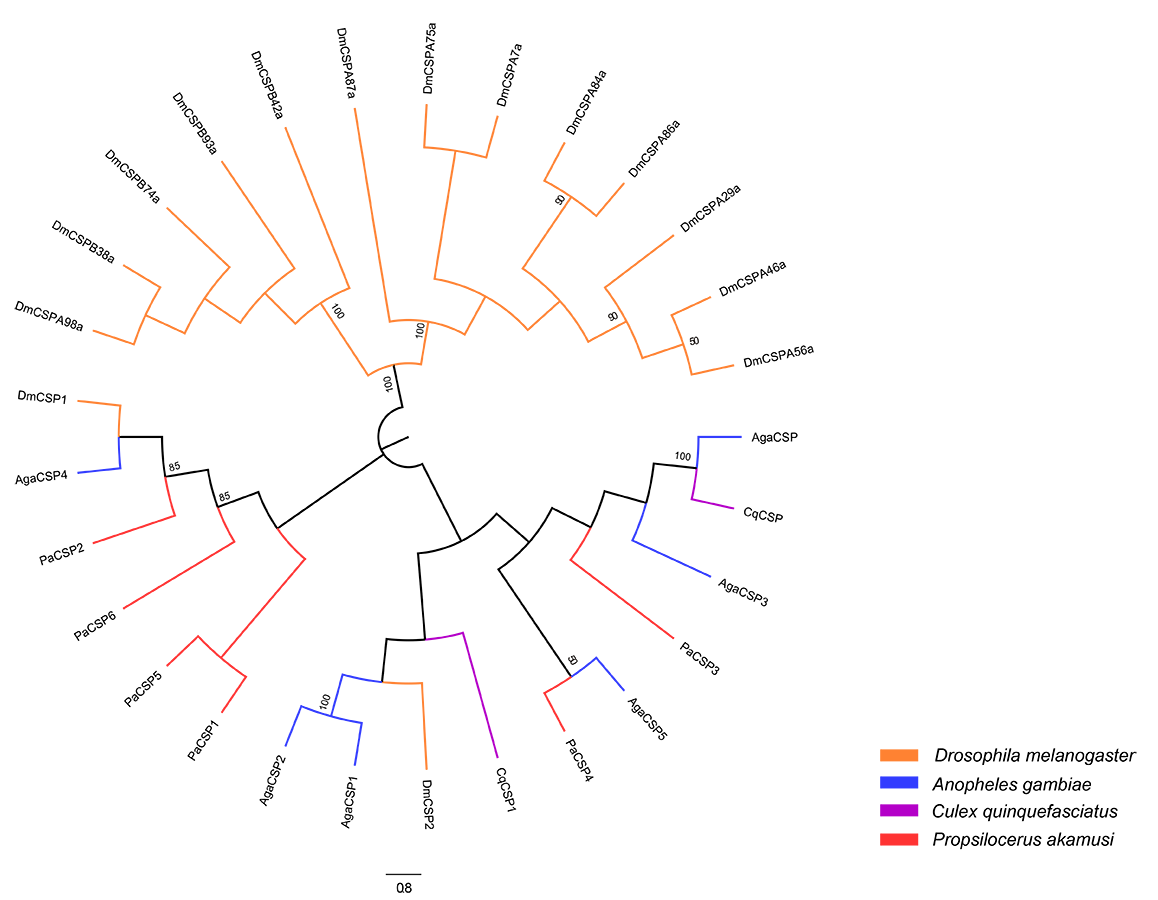

Supplement: Supplemental Information 16 — A. gambiae, C. quinquefasciatus and P. akamusi. Bootstrap values were calculated with 1,000 replicates and the values >50% are shown on the branches. Yellow: D. melanogaster; Blue: A. gambiae; Purple: C. quinquefasciatus; Red: P. akamusi. [file peerj-08-9584-s016.png]

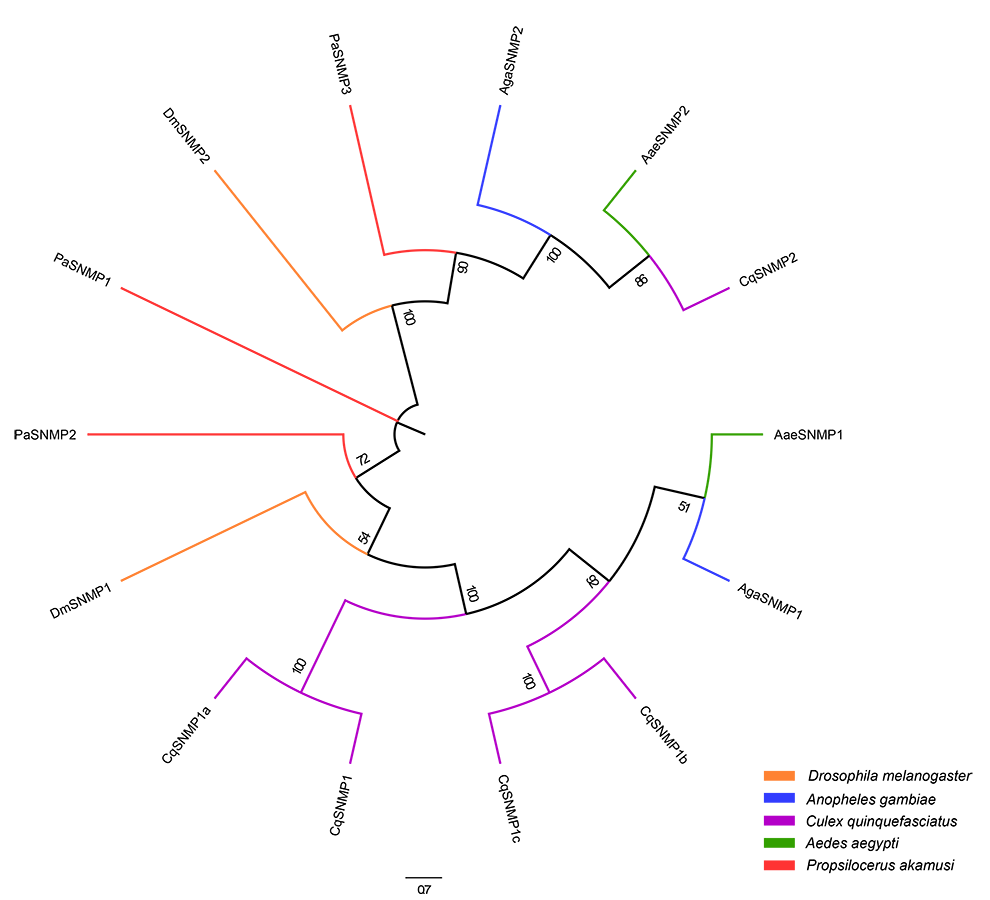

Supplement: Supplemental Information 17 — A. aegypti, A. gambiae, C. quinquefasciatus and P. akamusi. Bootstrap values were calculated with 1000 replicates and the values < 50% are shown on the branches. Yellow: D. melanogaster; Blue: A. gambiae; Purple: C. quinquefasciatus; Green: A. aegypti; Red: P. akamusi. [file peerj-08-9584-s017.png]
